# Temporal association between zolpidem medication and the risk of suicide: A 12-year population-based, retrospective cohort study

**DOI:** 10.1038/s41598-020-61694-9

**Published:** 2020-03-17

**Authors:** Chul-Hyun Cho, Hee-Jung Jee, Yoon-Ju Nam, Hyonggin An, Leen Kim, Heon-Jeong Lee

**Affiliations:** 1 Department of Psychiatry, School of Medicine, Chungnam National University, Daejeon, South Korea; 2 Department of Psychiatry, Sejong Chungnam National University Hospital, Sejong, South Korea; 3 Korea University Chronobiology Institute, Seoul, South Korea; 4 Department of Biostatistics, Korea University College of Medicine, Seoul, South Korea; 5 Department of Psychiatry, Korea University College of Medicine, Seoul, South Korea

**Keywords:** Psychiatric disorders, Drug regulation, Epidemiology

## Abstract

There have been concerns about abuse and unnecessary chronic administration of zolpidem, and zolpidem’s relation to suicide risk. To investigate the temporal association of zolpidem with the risk of suicide, we conducted a 12-year, population-based, retrospective cohort study on the National Health Insurance Service–National Sample Cohort (NHIS-NSC), South Korea. Data were collected from 2002 to 2013 from the NHIS-NSC, and data cleaning was performed for 1,125,691 subjects. Cox proportional hazards regression analysis was used to investigate the correlation over time between zolpidem medication and suicide. Over intervals commencing after 80 months of observation, the adjusted hazard ratio of suicides associated with the use of the zolpidem was 2.01 (95% CI: 1.58–2.56; p < 0.001). The mean cumulative number of days of zolpidem prescription was significantly longer in the suicide group than in the non-suicide group after log-transformation (p = 0.005). Cases of chronic use of zolpidem (over six months or one year) were significantly more common in the suicide group compared to the non-suicide group (p = 0.002 and 0.005, respectively). Subjects who received zolpidem medication had a significantly higher risk of suicide after at least 80 months of observation, suggesting a long-term increased suicide risk associated with insomnia exposed to zolpidem medication.

## Introduction

Insomnia has been loosely conceived as an insufficient amount of sleep or a characteristic poor quality of sleep, both of which are becoming more prevalent as modern society develops^1,2^. However, “insomnia” as rigorously defined currently is simply self-reported trouble falling or staying asleep or early awakening. Insomnia can generally be defined as having persistent difficulty with sleep initiation, duration, or quality, despite a sufficient environment and opportunities to sleep^3^. On the other hand, insufficient sleep is defined as a curtailed sleep pattern that has persisted, along with complaints of sleepiness during day^4^. Insomnia is not always accompanied by “insufficient sleep” that is objectively of less-than-normal duration nor is there always objectively poor sleep quality. Moreover, “insufficient sleep” can occur without insomnia, for example, when work duties do not allow sufficient time for sleep but the patient has no trouble falling or staying asleep. Insomnia is commonly associated with medical problems such as endocrine, cardiovascular, immune, and neoplastic disorders as well as with various circadian rhythm sleep disorders, with various mental problems such as disturbed mood, anxiety, irritability, concentration, and suicide, and with various environmental disturbances^5–8^. In recent years, the claim that insomnia should be interpreted as a comorbid disease that is independent of other mental disorders has led to a change in perspective with regard to insomnia, and argues for more active diagnostic analysis and therapeutic approaches^9^. Although the currently-preferred initial treatment for insomnia is cognitive-behavioral therapy^10^, hypnotics and sedatives are still being actively and widely prescribed as initial treatments for insomnia and for more poorly-characterized reasons^11^.

Hypnotics and sedatives can act as subjectively-effective treatment strategies for insomnia for at least a few weeks, though the long-term efficacy is debatable^12^. However, no medically-significant objective benefits have been documented, and taking hypnotics and sedatives could lead to serious medical and social issues owing to dependence, abuse, misuse, and various other side-effects of long-term medication^13–15^. Long-term use of hypnotics and sedatives is reportedly associated with the development of dementia or cancer^16–18^, and some studies have shown that these drugs are associated with increased mortality rates^18–20^. Reports of a strong association between sleeping pills and suicide are also being published^21–23^. Zolpidem is a very-commonly-prescribed non-benzodiazepine sleeping pill, one of the recently popular Z-drugs for treating insomnia^24^. Choi *et al*. reported that the zolpidem use group had a significantly higher risk of suicide about twice that of the control group, and dose-response relationships were observed from a population-based case-control study^25^.

Many benzodiazepine drugs used for insomnia are also used as anxiolytic agents, while insomnia is the sole indication for zolpidem^26^. One advantage of zolpidem is its short half-life and hence short duration of action, which makes drowsiness the following day less likely than with most alternatives; however, on the negative side, reports of suicide following zolpidem use are on the increase. One example is episodes of so-called complex behavior, characterized by an attempt at suicide with a bizarre mental state occurring with hours after consumption of the drug^23,27^. Epidemiological studies indicate further that zolpidem is associated with increased risk of suicide without regard to time of day of administration^23,28^. Suicide is one of the most crucial problems faced by society, with grave medical, economic, social, and cultural repercussions. South Korea is well known as the country with the highest suicide rates among member nations of the Organization for Economic Cooperation and Development^29^. Insomnia in South Korea continues to increase^30,31^, and the number of sleeping pills prescribed has also been on the rise^32^. As a hypnotic drug, zolpidem is easily accessible from both psychiatric clinics as well as from other clinical specialties^26^. In view of its widespread use, there have been concerns about abuse and misuse of zolpidem, and whether unnecessary chronic administration leads to deleterious effects^15,26^. Cognitive-behavioral therapy for insomnia (CBT-I) is more beneficial and is recommended as a standard treatment^33^. Zolpidem medication is not now the preferred treatment of insomnia, so excessively easy access to zolpidem is a problem that requires a solution^34,35^.

Based on previous clinical experience and epidemiological studies of the close association between zolpidem and suicide, it would be meaningful to investigate zolpidem in the special situation in South Korea. Although the previous study in Korea reported the relationship between zolpidem and suicide, it was limited to a case-control study^25^. Examining the temporal link between the exposure of zolpidem and the risk of suicide is a new and meaningful approach, although there are various limitations in the methodology and the interpretation. We aimed to investigate the temporal association of zolpidem consumption with the risk of suicide, by performing a population-based, retrospective cohort study on the National Health Insurance Service–National Sample Cohort (NHIS-NSC), South Korea^36^.

## Results

### The temporal association between zolpidem use and suicide: Cox proportional hazards regression analysis

**Figure 1:**
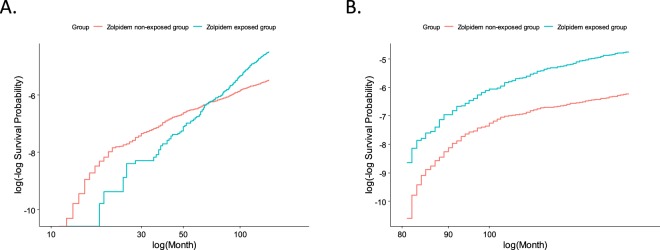
The graph of a Kolmogorov-type supremum test to assess the proportional hazard assumption to suicides between the zolpidem exposed group (ZEG) and zolpidem non-exposed group (ZNG). In verification of the proportional hazard assumption, the risk curves of the ZEG and the ZNG of the current data crossed at the 80-month time point, confirming that the proportional risk assumption had been violated (**A**). The proportional risk assumption is verified only on the second divided time-period of more than 80 months (**B**).

**Figure 2:**
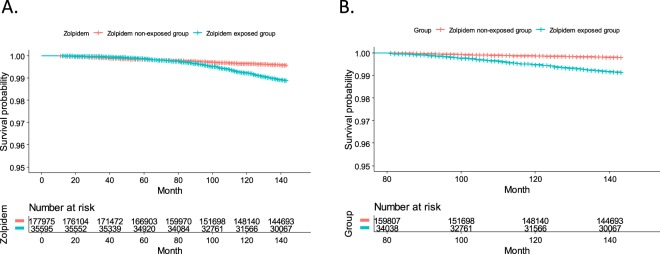
The graph of a Kaplan-Meier survival plot of suicide between the zolpidem exposed group (ZEG) and zolpidem non-exposed group (ZNG). Survival probability related only to suicides is presented between the ZEG and ZNG on whole time period (**A**) and the time period of more than 80 months (**B**).

When we conducted a verification of the proportional hazard assumption, the risk curves of the zolpidem exposed group (ZEG) and the zolpidem non-exposed group (ZNG) of the current data crossed at the 80-month time point, confirming that the proportional hazard assumption had been violated (Fig. 1A). Therefore, we divided the time-period into less than 80 months and more than 80 months, then applied Cox proportional regression model in each divided interval of cohort observation. Finally, we confirmed that the proportional risk assumption is verified only on the second divided time interval of more than 80 months (Fig. 1B). Moreover, a Kaplan-Meier survival plot of suicides for both groups was provided (Fig. 2).

We analyzed the relationship between zolpidem medication and suicide risk in two periods divided by 80 months. In the first divided time interval of <80 months, the hazard ratio of zolpidem exposure on suicide was not significant (Unadjusted = 1.14, p-value = 0.283; Adjusted = 0.83, p-value = 0.206) (Table 1 and Supplementary Table 1). For the analysis focusing on the second divided time interval of more than 80 months, cohort participants with less than 80 months of time to death or censoring were excluded from the analysis. Therefore, the number of observations used in this second-interval analysis was 194,993, and that of suicides was 590. When the confounding variables were adjusted in the second divided time interval of more than 80 months, the suicide hazard ratio for zolpidem prescription of ≥28 days was almost twice (Unadjusted = 4.32, p-value < 0.001; Adjusted = 2.01, p-value < 0.001) (Table 1 and Supplementary Table 2).

**Table 1:** The temporal association between zolpidem medication and the risk of suicide in the two divided time-period intervals (less than 80 months and more than 80 months from the date of the initial exposure of the zolpidem exposed group (ZEG)): Cox proportional hazards regression analysis.

Variable		Unadjusted HR				Adjusted HR			
		HR	95% C.I		P-value	HR	95% C.I		P-value
Zolpidem medication	> 80 months	4.32	3.67	5.09	<0.001	**2.01**	**1.58**	**2.56**	**<0.001**
	$\le$ 80 months	1.14	0.90	1.44	0.283	0.83	0.61	1.11	0.206

The two time-period intervals (less than 80 months and more than 80 months from the date of the initial exposure of the zolpidem exposed group (ZEG)) were divided according to the verification of the proportional hazard assumption. Cox proportional hazards regression analyses were used to investigate the temporal correlation between zolpidem medication and the risk of suicide with statistical control of confounding factors. Using univariate Cox proportional hazards regression analyses, significant variables were selected for analysis as potential confounders at a significance level of 0.1 (Unadjusted Hazard Ratio (HR)). The study of all variables can be found in detail in the Supplementary Tables 1 and 2.

### Zolpidem chronic prescription in relation to suicide

As shown in Table 2, the mean cumulative zolpidem prescription days was 222.52 days (SD: 381.12) and the median was 69 days in the suicide group; the mean cumulative zolpidem prescription days was 191.56 days (SD: 431.43) and median 60 days in the control non-suicide group. We performed the independent two-sample t-test between suicide and control non-suicide groups after log-transform because of the right-skewed distribution of cumulative prescription days. We found that the mean cumulative zolpidem prescription days was significantly longer in the suicide group than in the control group (p = 0.0053).

**Table 2:** The comparison of the mean cumulative zolpidem prescription days between suicide and non-suicide groups.

Groups	number of subjects (N)	mean	SD	median	minimum	maximum	p-value^†^
Suicide group	361	222.52	381.12	69	3	2819	0.005
Non-suicide group	35234	191.56	431.43	60	2	26177	

^†^p-value: the statistical significance by independent two-sample t-test after log-transform.

Next, we examined the frequency of chronic zolpidem medication between the suicide and the control groups according to the cumulative prescription duration of 6 months or one year during whole cohort period, respectively (Table 3). When the cumulative prescription period of 6 months or more was defined as chronic medication group, the suicide rate in the chronic medication group (1.32%) was significantly higher than the suicide rate in the control (no chronic medication) group (0.93%) from the chi-square test (p = 0.002). When the cumulative prescription period of one year or more was defined as chronic medication group, the rate of suicide was 1.41% in the chronic medication group and 0.96% in the control group, showing statistical significance (p = 0.005). It was found that the suicide rate was significantly more common in the chronic zolpidem exposure subjects (whether over 6 months or one year) compared to the control group.

**Table 3:** The comparison of the frequency of the chronic zolpidem medication group between suicide and control groups according to the cumulative prescription duration of 6 months or one year, respectively.

Chronic zolpidem medication exposure: the cumulative prescription duration (${\rm{\ge }}$ 6 months or ${\rm{\ge }}$ one year)	Suicide group (N (%))	Non-suicide group (N (%))	P-value
**The cumulative zolpidem prescription duration** ${\rm{\ge }}$ **6 months**			
Yes	103 (1.32%)	7708 (98.68%)	0.002
No	258 (0.93%)	27526 (99.07%)	
**The cumulative zolpidem prescription duration** ${\rm{\ge }}$ **one year**			
Yes	62 (1.41%)	4339 (98.59%)	0.005
No	299 (0.96%)	30895 (99.04%)	

## Discussion

In this study, it was confirmed that the use of zolpidem was not significantly associated with the risk of suicide during a 12-year retrospective period. However, when a subject is over a certain period of time (more than 80 months) after taking zolpidem, the risk of suicide of the ZEG is about twice that of the ZNG.

The association between zolpidem medication and suicide can be considered in three main respects as below: firstly, focusing on death due to zolpidem during an overdose taken to commit suicide; secondly, suicide as a part of the paradoxical behavior just after zolpidem administration; and thirdly, the increased risk of suicide in the zolpidem exposure group^21,23,37–40^. The present study is for the third of these, i.e. analyzing the effect of zolpidem exposure on the overall risk of suicide. Previous studies on this subject were mostly about the association of suicide with zolpidem as a cross-sectional analysis^14,23,28^, however the present study is characterized by a retrospective cohort analysis based on the 12-year data of the NHIS-NSC, which attempted to investigate zolpidem medication as a causal factor of suicide risk.

Insomnia is a relatively common condition with an increasing prevalence rate in modern society^33^. If the dosage and duration of zolpidem are appropriately prescribed as a treatment for some types and stages of insomnia, some authors feel it is an acceptable treatment option^24^. It has been argued that in the United States (and perhaps elsewhere), most patients prescribed zolpidem does not have a diagnosis of insomnia or have multiple contraindications^17^. However, due to insufficient standard treatment based on cognitive-behavioral therapy for insomnia (CBT-I) in the actual clinic setting, excessive demand of hypnotics by patients, and indifference of therapists, the hypnotic medication including Z-drug may cause problems such as abuse, tolerance, withdrawal, dependence, and unnecessary chronic use of medication^38,41,42^. The long-term effects of zolpidem medication have been reported previously, such as the incidence of cancer and the mortality, suggesting that zolpidem medication is generally associated with adverse effects on health^43–45^.

In this study, we found that subjects who received zolpidem medication had a significantly higher risk (Risk ratio ≈ 2) of suicide after about 80 months beyond the first zolpidem treatment. Although the interpretation of the results of the present study is somewhat limited, they suggest that patients who have received clinically significant cumulative doses of zolpidem (more than 28 days of zolpidem in a year) show an increased risk of suicide after about 80 months of first zolpidem exposure. This is noteworthy in that the results of the long-term association of zolpidem medication with suicide risk are hereby presented. We could speculate that some characteristics associated with zolpidem medication or some characteristics of those patients treated with zolpidem medication are closely related to suicide and may significantly increase the risk of suicide after a certain period of time by a mechanism associated with these characteristics.

First, we could consider that certain features of treatment using zolpidem may increase the risk of suicide. In general, about 2/3 to 3/4 of patients who take hypnotic medication continue prolonged therapy, and these patients become psychologically habituated or physically addicted to hypnotics and fear withdrawal, even though studies show that such patients benefit from withdrawal, not from prolonged therapy^14,42^. This may be because the insomnia is not properly controlled, or it may be because insomnia patients did not receive effective and appropriate treatment for the condition in the clinics. The results of this study showed that the risk of suicide significantly increased after more than about 6.7 years (80 months) of the administration of zolpidem. It can be inferred that the attributes leading to the long-term use of zolpidem had an impact on the outcome of this study. In the present study, we could confirm the difference in the cumulative zolpidem dosing days and the difference in the number of long-term zolpidem dosing groups between the suicide and non-suicide groups. As a result, it was found that the cumulative number of zolpidem dosing days was significantly longer, and the number of long-term zolpidem dosing groups were both significantly higher in the suicide group. The long-term dosing of zolpidem, which is more frequent in the suicide population, is notable. This may be an indirect basis that might explain the significant increase in the risk of suicide after 6.7 years of administration of zolpidem, which is a key result of this study.

Next, we would consider that certain characteristics of patients taking zolpidem may be closely related to suicide. The sole indication for zolpidem prescribing is insomnia, and people with insomnia are more likely to have a variety of psychiatric disorders, including major depressive disorders, bipolar disorders, and anxiety disorders^46–48^. Therefore, when interpreting the results, the mental health of those taking zolpidem should be fully considered. This is because mental health problems can increase the risk of suicide. In this study, we took into account the variables (including psychiatric disorders) that might have an influence on suicide risk. Based on the preliminary analysis, we statistically adjusted the factors that might increase the risk of suicide in patients being treated with zolpidem. However, since it is impossible to measure and take into account all other factors related to suicide in the real world, it is necessary to take this fact into consideration when carrying out the research and interpreting the results, no matter how thorough and detailed the statistical analysis performed. If future prospective cohort studies are undertaken, data on more comprehensive and detailed variables associated with zolpidem medication and suicide will be available.

Consideration should be given as to why the risk of suicide has not been significantly increased within about seven years after zolpidem administration. First, insomnia often progresses to chronic insomnia, and presumably, long-term exposure of zolpidem may be closely related to chronic insomnia^49,50^. Therefore, it can be speculated that chronic insomnia is associated with a high suicide risk after a relatively long-term period. Second, we must consider that suicide attempts could not be measured in the present study. In this study, only suicide death data could be obtained. However, in the case of suicide, previous suicide attempts are frequent, and the prior suicide attempt history also increases the risk of suicide^51,52^. It should be taken into account that the suicide risk may be missed due to the absence of suicide attempts data. Third, chronic insomnia is often accompanied by a variety of psychological difficulties, such as depression and anxiety^53,54^. Although not confirmed by diagnosis, it can be speculated that mental burden accumulated over a long period of time due to chronic insomnia can increase the suicide risk after a certain period. It should be noted that these considerations are only speculations to illustrate the temporal associations of this study and that verification is necessary through future research.

This study has some limitations. Firstly, we performed a retrospective cohort analysis. Although a prospective clinical trial or a prospective cohort study may be the ideal way to determine the association between zolpidem and suicide, there are ethical restrictions to research designed to conduct prospective studies on the characteristics of suicide-related matters. Second, the mechanism of how zolpidem medication increases the risk of suicide is unknown. This is a blind spot for most large-scale retrospective cohort studies. It is necessary to study in detail various methodologies in order to elucidate the factors that mediate the effects of zolpidem on suicide. Nevertheless, in this study, it is meaningful in that a temporal relationship was suggested between zolpidem medication and suicide for a large sample. Third, there is a lack of reliable information on the cumulative effect of zolpidem exposure on the risk of suicide. In particular, it may be worthwhile to investigate the cumulative effect of zolpidem medication. In view of the retrospective design of this study, it was difficult to reach any tangible conclusions on this point. However, in order to compensate for this, the number of days of cumulative zolpidem medication and the distribution of the long-term zolpidem medication group were analyzed in the present study. Fourth, the type of registration information obtained from NHIS-NSC’s database is very limited. Suicide is influenced by a variety of factors, including socioeconomic factors, cultural factors, individual support groups, physical health, and psychological conditions. Psychological problems can be helped through psychiatric care, but are sometimes handled by psychologists. Various factors, such as cultural factors, lifestyle, and substance use patterns, are difficult to measure and cannot be obtained from this database. Because of this limitation, confounding factors for detailed analysis could not be fully considered. The number of unmeasured confounding variables is a vulnerable point of this study, and therefore, we should be careful when interpreting the results. Fifth, diagnosed anxiety was controlled statistically for participants receiving zolpidem, but it was not determined if the diagnosis of anxiety preceded or followed the use of zolpidem. It has been reported that zolpidem withdrawal may lead to anxiety, and the design does not distinguish whether anxiety might in some cases be a result of zolpidem prescription rather than a cause thereof.

This study has several strengths. Firstly, this study is an analysis using NHIS-NSC which contains various national health insurance data. The NHIS-NSC is a cohort with well-refined data quality, while being representative of the general population. Secondly, the 12-year retrospective cohort study overcame the limitation of cross-sectional studies. Cross-sectional studies may have their own merits, but their limitations are clear in that they cannot distinguish temporal relations. In this study, we analyzed the suicide risk of subjects exposed to zolpidem for 12 years and found that zolpidem exposure was associated with increased risk of suicide and it was significant at some time point, suggesting a temporal relation. Thirdly, the analysis was carried out through clinically more appropriate and reliable operational definition. As we proceeded with our research, we were concerned about how well the operational definition could be reflected in the clinical setting for a retrospective cohort study. We thought that it was not an optimal method to simply include all the subjects who were exposed to zolpidem for just a few days.

All drugs have advantages and benefits on the one hand, as well as disadvantages and pitfalls on the other. Therefore, a well-known “clinical pearl” is to always weigh up the negative features of therapy against the severity of the effects of the potentially untreated disease before prescribing a drug. Usually, the adverse effects of the medication are caused by the pharmacodynamic and/or pharmacokinetic effects of the drug itself, and also by the interaction with other drugs. Moreover, we must think about the long-term adverse associations of drugs, as well as the short-term effects. In particular, if the drug is likely to be abused or misused, as opposed to the actual purpose, or if the drug is likely to require long-term administration, then the clinician should consider long-term associations. In this respect, the present study can be used as a shred of valuable evidence for the long-term use of zolpidem. Although this study does not identify causality or confirm the exact mechanism of temporal association, it is clinically applicable that a significant association between zolpidem exposure and suicide risk is observed after a long period of time. In the future, conducting research based on advanced study design should identify the causal relationship between zolpidem administration and suicide and its mechanism. Above all, it was once again confirmed that it is most important to reduce chronic insomnia, which would require long-term administration of sleeping pills such as zolpidem, through effective and standard treatments for insomnia in actual clinical practice.

## Methods

### Data sources

The NHIS-NSC is a population-based cohort in South Korea, developed to provide public health researchers with representative information regarding utilization by citizens of the NHIS, which is a universal-coverage health insurance system for all citizens. Cohort participants were randomly sampled from the 2002 Korean nationwide health insurance database until 2013. The NHIS-NSC contains personal information (gender, residential area, type of health insurance, level of income, type and grade of disability registered, and birth and death) and medical treatment data (participants’ medical bills claimed by medical service providers, medical care institution information regarding the type of institution, establishment, location, number of beds, facilities, and physicians, and pharmacy information) for Korean citizens, who were categorized as insured employees, insured self-employed individuals or medical aid beneficiaries. The standard detailed information on the NHIS-NSC is described in the previous cohort profile paper^36^. Cohort follow-up data were collected from January 2002 to December 2013 from NHIS-NSC, and data cleaning was performed using a database of drug prescriptions and diagnosis data for 1,125,691 patients over 12 years. Participants under the age of 10 years definitions from the current analyses. Finally, a retrospective cohort analysis was conducted on 888,739 participants to investigate the association of zolpidem prescription with suicide.

The ZEG was defined as having a cumulative number of days of prescribed zolpidem exposure of 28 days or more within one year (365 consecutive days) from the initial administration of zolpidem during the 12 years for the cohort. In Korea, the national health insurance system limits the continuous prescription of zolpidem to 28 days per prescription, which is a national policy to discourage longer-term use and thus to avoid dependence or abuse. In addition, because the investigators thought that either short-term or low-dose administration of zolpidem might not produce sufficient adverse effects to be measurable, the inclusion threshold for the ZEG was assigned if cumulative dosing days were 28 days or more within one year (365 consecutive days). The size of the sample in the ZEG was 35,823. In order to ensure that study subjects were “clean (zolpidem-free at the beginning of cohort observation)”, a period of two months from the cohort’s starting time-point was defined as a washout period. If the participants were exposed to zolpidem during the first two months of the washout period, we excluded them from the analyses lest the effects of zolpidem on suicide may have been distorted, because we did not have information about previous zolpidem exposure before the subjects were entered into the cohort. The final size of the ZEG was 35,595. The ZNG who was not exposed to zolpidem during the study period. The control ZNG group was selected to match the ZEG by gender and age in a 1:5 ratio, and finally 177,975 control subjects were included (Supplementary Table 3). Therefore, the total size of the sample analyzed in the cohort was 213,570.

In this data from NHIS-NSC, information about suicide attempts was inaccurate and unsatisfactory, so we investigated only those suicides that were actually completed. The inclusion criteria of the “suicide group” was set to be cases in which the code of Korean Standard Classification of Diseases (KCD) associated with suicides (X60~X84) and the date of death existed at the same time in sample data. Finally, 2,815 people were selected that met the criteria of the suicide group in the total observation of 12 years.

This study was approved by the NHIS inquiry commission for using the data from NHIS-NSC. The requirement to obtain written consent was waived due to the retrospective design, and anonymity of the national insurance claims data was maintained for the protection of the personal privacy of participants. For the focused analysis of the impact of zolpidem medication on the suicide rates from the specific data derived from NHIS-NSC, we obtained approval by the Institutional Review Board of Korea University Anam Hospital (ED15238); the study was conducted in accordance with the Declaration of Helsinki.

### Statistical analysis

We conducted a multivariate Cox proportional hazards regression with control of confounding variables to investigate the temporal correlation over time between the zolpidem medication and suicide with statistical control of confounding factors^55^. Significant variables were selected for analysis as potential confounders at a significance level of 0.1 (Unadjusted Hazard Ratio (HR)).

The potentially-confounding factors were chosen for analysis in the cohort. National health insurance member classification (the self-employed insured – householder, house member; the employee – insured, dependent; and medical aid beneficiaries – householder, house member), income distribution (from the first decile to the tenth decile), benzodiazepine drug medication (alprazolam, bromazepam, chlordiazepoxide, clonazepam, diazepam, etizolam, lorazepam, and triazolam), major medical diseases (malignant neoplasm, cerebrovascular and cardiovascular diseases, pneumonia, diabetes, chronic lower respiratory diseases, liver diseases, and hypertensive diseases), psychiatric disorders (schizophrenic spectrum disorders, major depressive disorders, bipolar disorders, anxiety disorders, substance use disorders, insomnia disorders, and other psychiatric disorders) were considered as the candidate confounding factors.

The event of interest, censoring, and time to the event were defined as follows. The event of interest was defined as “death due to suicide” and the censoring variable was defined as “end of study observation” in December 2013 or “death due to reasons other than the event of interest.” As for the time to an event, this was defined as “the time from the first entry into the cohort to the time of the event of interest (death due to suicide) or censoring”.

To confirm the assumption that the hazard ratio does not depend on time needs to be verified, we conducted a Kolmogorov-type supremum test using cumulative sums of martingale residuals and graphics to assess the proportional hazards assumption^56^. We divided the groups according to ZEG or ZNG and plotted the log (-log (survival)) curves with the Kaplan-Meier survival analysis. If the survival curves crossed each other, it could be deduced that the assumption of proportional risk had been violated, and then the additional analysis would be needed, e.g., dividing the overall interval at the time when the curves intersected. Additional analyses were performed to confirm the degree of concordance of long-term zolpidem with suicide. We analyzed the cumulative prescription period of the ZEG between suicide and non-suicide groups accordingly. The parameter ‘The cumulative zolpidem prescription days’ was defined as the actual cumulative prescription period in a number of days from the date of the first zolpidem prescription to the first day of “the zolpidem prescription discontinuation.” “The zolpidem prescription discontinuation” was defined as the situation where zolpidem was not prescribed for more than 1 consecutive year. The cumulative zolpidem prescription days were compared between the suicide and control (non-suicide) groups. In addition, the suicide rate in “the chronic zolpidem medication group (the cumulative prescription days at 6 months or one year)” was investigated.

All analyses were performed using SAS 9.4 (SAS Institute Inc., Cary, NC, USA) program and the survival plot used the package survival of the R program (Ver 3.4.4).

## Supplementary information

Supplementary Tables.

## Data Availability

The datasets used and/or analyzed during the current study are not available from the corresponding author, because the dataset was obtained from the National Health Insurance Service–National Sample Cohort, South Korea.
