# Supplementary material for: Temporal association between zolpidem medication and the risk of suicide: A 12-year population-based, retrospective cohort study
Source: Sci Rep. 2020 Mar 17;10:4875. doi: 10.1038/s41598-020-61694-9 (PMC7078307; doi:10.1038/s41598-020-61694-9)
Supplement: Supplementary file 1 — Supplementary Tables. [file 41598_2020_61694_MOESM1_ESM.pdf]

Title Page

Supplementary Tables

Temporal association between zolpidem medication and the risk of suicide: A 12-year population-based, retrospective cohort study

Chul-Hyun Cho, M.D., Ph.D.[1,2,3], Hee-Jung Jee, Ph.D.[4], Yoon-Ju Nam, M.D.[5], Hyonggin An, Ph.D.[4], Leen Kim, M.D., Ph.D.[3,5], Heon-Jeong Lee, M.D., Ph.D.[3,5*]

[1]*Department of Psychiatry, School of Medicine, Chungnam National University, Daejeon, South Korea*
[2]*Department of Psychiatry, Sejong Chungnam National University Hospital, Sejong, South Korea*
[3]*Korea University Chronobiology Institute, Seoul, South Korea*
[4]*Department of Biostatistics, Korea University College of Medicine, Seoul, South Korea*
[5]*Department of Psychiatry, Korea University College of Medicine, Seoul, South Korea*

Running title: Zolpidem and suicide risk

[*]Corresponding author: Heon-Jeong Lee, MD, PhD, Department of Psychiatry, Korea University College of Medicine, 73 Inchon-ro, Seongbuk-gu, Seoul 02841, Republic of Korea, Tel.: +82-2-920-6721, Fax: +82-2-929-7679, E-mail: leehjeong@korea.ac.kr

Supplementary Table 1. The association between zolpidem medication and the suicide risk in the time interval less than 80 months from the date of the initial exposure of the zolpidem exposed group (ZEG): Cox proportional hazards regression analysis.

	Unadjusted HR				Adjusted HR			
Variable	HR	95% C.I		P-value	HR	95% C.I		P-value
**Zolpidem exposure**	1.14	0.9	1.44	.283	0.83	0.61	1.11	.206
**National health insurance member classification**				<.001				<.001
the self-employed insured house member	0.48	0.36	0.64	<.001	0.48	0.36	0.65	<.001
the employee insured	0.42	0.3	0.6	<.001	0.39	0.27	0.55	<.001
the employee dependent	0.85	0.68	1.06	0.139	0.93	0.74	1.17	0.536
the medical aid beneficiaries householder	1.31	0.86	1.98	0.21	1.2	0.74	1.95	0.463
the medical aid beneficiaries house member	0.69	0.28	1.68	0.41	0.61	0.24	1.55	0.299
the self-employed insured householder (reference)								
**Income decile group**				0.026				0.126
Decile 1	0.96	0.61	1.5	0.861	1.65	1.13	2.4	0.01
Decile 2	0.75	0.46	1.22	0.245	1.21	0.79	1.85	0.374
Decile 3	0.81	0.51	1.29	0.376	1.3	0.87	1.95	0.198
Decile 4	0.55	0.33	0.91	0.019	0.9	0.58	1.4	0.64
Decile 5	0.54	0.33	0.88	0.014	0.89	0.58	1.38	0.608
Decile 6	0.65	0.41	1.03	0.065	1.09	0.73	1.62	0.672
Decile 7	0.61	0.39	0.97	0.036	1.03	0.69	1.52	0.9
Decile 8	0.55	0.35	0.88	0.012	0.95	0.65	1.4	0.803
Decile 9	0.58	0.37	0.91	0.017	0.99	0.69	1.43	0.97
Decile 10	0.58	0.38	0.9	0.014	.	.	.	.
Decile 0 (reference)								
**Sedatives-Hypnotics**								
Alprazolam	1.41	1.15	1.74	0.001	1.69	1.3	2.21	<.001
Bromazepam	2.25	1.48	3.42	<.001	1.89	1.2	2.97	0.006
Chlordiazepoxide	2.55	1.27	5.13	0.009	1.55	0.74	3.24	0.242
Clonazepam	1.48	1.01	2.15	0.042	1.07	0.7	1.62	0.772
Diazepam	1.06	0.87	1.29	0.571	.	.	.	.
Etizolam	0.57	0.35	0.92	0.021	0.57	0.35	0.94	0.027
Lorazepam	1.7	1.26	2.29	<.001	1.14	0.79	1.65	0.473
Triazolam	2.36	1.76	3.15	<.001	2.28	1.61	3.24	<.001
**Major medical diseases**								
Malignant neoplasm	0.7	0.54	0.91	0.008	0.85	0.65	1.13	0.262
Cardiovascular diseases	0.79	0.64	0.98	0.029	1.03	0.82	1.31	0.781
Cerebrovascular diseases	0.81	0.65	1.02	0.073	0.96	0.74	1.23	0.726

Pneumonia	0.5	0.38	0.65	<.001	0.66	0.5	0.88	0.004
Diabetes	0.87	0.72	1.05	0.139	.	.	.	.
Chronic lower respiratory diseases	0.37	0.31	0.44	<.001	0.42	0.34	0.51	<.001
Liver diseases	0.46	0.38	0.56	<.001	0.49	0.4	0.6	<.001
Hypertensive diseases	1.01	0.84	1.22	0.887	.	.	.	.
**Psychiatric disorders**								
Schizophrenic spectrum disorders	1.95	1.33	2.85	<.001	1.38	0.91	2.11	0.135
Major depressive disorders	1.25	1.03	1.52	0.026	1.43	1.11	1.84	0.005
Bipolar disorders	1.07	0.64	1.8	0.785	.	.	.	.
Anxiety disorders	0.82	0.68	0.98	0.033	0.92	0.73	1.15	0.452
Substance use disorders	2.67	1.86	3.82	<.001	2.18	1.46	3.26	<.001
Insomnia disorders	1	0.83	1.21	0.986	.	.	.	.
Other mental disorders	0.77	0.59	0.99	0.038	0.65	0.49	0.86	0.003

Cox proportional hazards regression analyses were used to investigate the correlation over time between the zolpidem medication and suicide with statistical control of confounding factors. Using univariate Cox proportional hazards regression analyses, significant variables were selected for analysis as potential confounders at a significance level of 0.1 (Unadjusted Hazard Ratio (HR)).

Supplementary Table 2. The association between zolpidem medication and the suicide risk in the time interval more than 80 months from the date of the initial exposure of the zolpidem exposed group (ZEG): Cox proportional hazards regression analysis.

	Unadjusted HR				Adjusted HR			
Variable	HR	95% C.I		P-value	HR	95% C.I		P-value
**Zolpidem exposure**	4.32	3.67	5.09	<.001	**2.01**	**1.58**	**2.56**	**<.001**
**National health insurance member classification**				<.001				<.001
the self-employed insured house member	0.54	0.43	0.69	<.001	0.58	0.46	0.74	<.001
the employee insured	0.41	0.3	0.56	<.001	0.47	0.34	0.64	<.001
the employee dependent	0.72	0.59	0.88	.002	0.78	0.63	0.95	.016
the medical aid beneficiaries householder	1.21	0.81	1.8	.35	1.25	0.8	1.95	.33
the medical aid beneficiaries house member	0.63	0.28	1.42	.268	0.62	0.27	1.43	.264
the self-employed insured householder (reference)								
**Income decile group**				.019				.07
Decile 1	0.99	0.65	1.5	.943	1.47	1.05	2.05	.023
Decile 2	0.76	0.48	1.2	.237	1.07	0.74	1.56	.71
Decile 3	0.73	0.47	1.15	.172	1.05	0.73	1.51	.801
Decile 4	0.46	0.28	0.76	.002	0.68	0.45	1.03	.069
Decile 5	0.62	0.4	0.98	.038	0.92	0.64	1.32	.647
Decile 6	0.66	0.43	1.02	.06	1	0.71	1.41	.989
Decile 7	0.56	0.36	0.87	.009	0.84	0.59	1.19	.326
Decile 8	0.69	0.45	1.04	.076	1.07	0.78	1.48	.666
Decile 9	0.61	0.4	0.92	.018	0.93	0.68	1.28	.657
Decile 10	0.7	0.47	1.03	.071	.	.	.	.
Decile 0 (reference)								
**Sedatives-Hypnotics**								
Alprazolam	2.9	2.45	3.42	<.001	1.45	1.18	1.79	.001
Bromazepam	3.75	2.79	5.04	<.001	1.49	1.09	2.04	.013
Chlordiazepoxide	5.18	3.31	8.09	<.001	1.36	0.85	2.18	.198
Clonazepam	3.53	2.8	4.46	<.001	1.09	0.83	1.42	.544
Diazepam	1.67	1.41	1.97	<.001	0.94	0.78	1.15	.551
Etizolam	1.98	1.54	2.54	<.001	1.14	0.88	1.49	.316
Lorazepam	4.29	3.53	5.21	<.001	1.24	0.97	1.58	.082
Triazolam	4.49	3.65	5.54	<.001	1.51	1.19	1.93	.001
**Major medical diseases**								
Malignant neoplasm	1.45	1.19	1.75	<.001	1.16	0.95	1.43	.144
Cardiovascular diseases	1.26	1.06	1.49	.011	0.91	0.74	1.11	.33
Cerebrovascular diseases	1.46	1.23	1.75	<.001	1.08	0.88	1.32	.462
Pneumonia	1.19	0.99	1.42	.071	0.99	0.81	1.2	.901

Diabetes	1.43	1.21	1.68	<.001	1.1	0.91	1.34	.326
Chronic lower respiratory diseases	0.84	0.71	1	.051	0.64	0.53	0.77	<.001
Liver diseases	1.21	1.02	1.44	.027	0.8	0.66	0.97	.023
Hypertensive diseases	1.24	1.05	1.47	.01	0.92	0.75	1.13	.432
**Psychiatric disorders**								
Schizophrenic spectrum disorders	4.59	3.59	5.85	<.001	1.54	1.16	2.05	.003
Major depressive disorders	3.05	2.59	3.6	<.001	1.57	1.26	1.95	<.001
Bipolar disorders	4.51	3.53	5.76	<.001	1.32	0.99	1.77	.059
Anxiety disorders	1.79	1.5	2.12	<.001	0.94	0.75	1.16	.548
Substance use disorders	6.44	5.13	8.09	<.001	2.61	2.01	3.39	<.001
Insomnia disorders	2.91	2.45	3.46	<.001	1.24	0.97	1.6	.09
Other mental disorders	1.59	1.32	1.91	<.001	0.72	0.59	0.89	.002

Cox proportional hazards regression analyses were used to investigate the correlation over time between the zolpidem medication and suicide with statistical control of confounding factors. Using univariate Cox proportional hazards regression analyses, significant variables were selected for analysis as potential confounders at a significance level of 0.1 (Unadjusted Hazard Ratio (HR)).

Supplementary Table 3. Basic information of zolpidem exposed group (ZEG) and zolpidem non-exposed group (ZNG).

	Zolpidem exposed group (N=35,595)		Zolpidem non-exposed group (N=177,975)		p value
**Gender (N(%))**					>.9999
Male	14,051	39.47	70,255	39.47	
Female	21,544	60.53	107,720	60.53	
**Age group (N(%))**					>.9999
10s					
10-14 years old	394	1.11	1,970	1.11	
15-19 years old	790	2.22	3,950	2.22	
20s					
20-24 years old	1,308	3.67	6,540	3.67	
25-29 years old	1,471	4.13	7,355	4.13	
30s					
30-34 years old	1,968	5.53	9,840	5.53	
35-39 years old	2,356	6.62	11,780	6.62	
40s					
40-44 years old	3,586	10.07	17,930	10.07	
45-49 years old	3,574	10.04	17,870	10.04	
50s					
50-54 years old	3,391	9.53	16,955	9.53	
55-59 years old	3,381	9.5	16,905	9.5	
60s					
60-64 years old	4,325	12.15	21,625	12.15	
65-69 years old	3,869	10.87	19,345	10.87	
70s					
70-74 years old	2,774	7.79	13,870	7.79	
75-79 years old	1,535	4.31	7,675	4.31	
More than 80s					
80-84 years old	668	1.88	3,340	1.88	
≥ 85 years old	205	0.58	1,025	0.58	
**Suicide (N(%))**					<.0001
Yes	35,234	98.99	177,307	99.62	
No	361	1.01	668	0.38	
**National health insurance member classification (N(%))**					<.0001
the self-employed insured householder	8,983	25.24	42,897	24.1	
the self-employed insured house member	8,633	24.25	41,815	23.49	
the employee insured	4,346	12.21	26,332	14.8	
the employee dependent	12,033	33.81	58,513	32.88	
the medical aid beneficiaries householder	1,137	3.19	6,325	3.55	
the medical aid beneficiaries house member	463	1.3	2,093	1.18	
**Income decile group (N(%))**					<.0001
Decile 0	1,600	4.5	8,418	4.73	

Decile 1	2,665	7.49	13,464	7.57	
Decile 2	2,276	6.39	11,611	6.52	
Decile 3	2,564	7.2	12,522	7.04	
Decile 4	2,537	7.13	13,658	7.67	
Decile 5	2,860	8.03	14,709	8.26	
Decile 6	3,099	8.71	16,187	9.1	
Decile 7	3,403	9.56	18,036	10.13	
Decile 8	3,814	10.71	20,382	11.45	
Decile 9	4,517	12.69	23,224	13.05	
Decile 10	6,260	17.59	25,764	14.48	
**Sedatives-Hypnotics (N(%))**					
Alprazolam: Yes	17,386	48.84	23,043	12.95	<.0001
Alprazolam: No	18,209	51.16	154,932	87.05	
Bromazepam: Yes	2,615	7.35	2,176	1.22	<.0001
Bromazepam: No	32,980	92.65	175,799	98.78	
Chlordiazepoxide: Yes	765	2.15	691	0.39	<.0001
Chlordiazepoxide: No	34,830	97.85	177,284	99.61	
Clonazepam: Yes	5,576	15.67	3,498	1.97	<.0001
Clonazepam: No	30,019	84.33	174,477	98.03	
Diazepam: Yes	19,052	53.52	46,273	26	<.0001
Diazepam: no	16,543	46.48	131,702	74	
Etizolam: Yes	5,373	15.09	7,886	4.43	<.0001
Etizolam: No	30,222	84.91	170,089	95.57	
Lorazepam: Yes	8,449	23.74	5,073	2.85	<.0001
Lorazepam: No	27,146	76.26	172,902	97.15	
Triazolam: Yes	7,433	20.88	3,267	1.84	<.0001
Triazolam: No	28,162	79.12	174,708	98.16	
Zolpidem: Yes	35,595	100	-	-	<.0001
Zolpidem: No	-	-	177,975	100	
**Major medical diseases (N(%))**					
Malignant neoplasm: Yes	9,554	26.84	31,048	17.45	<.0001
Malignant neoplasm: No	26,041	73.16	146,927	82.55	
Cardiovascular diseases: Yes	15,026	42.21	45,825	25.75	<.0001
Cardiovascular diseases: No	20,569	57.79	132,150	74.25	
Cerebrovascular diseases: Yes	13,309	37.39	38,687	21.74	<.0001
Cerebrovascular diseases: No	22,286	62.61	139,288	78.26	
Pneumonia: Yes	12,589	35.37	40,566	22.79	<.0001
Pneumonia: No	23,006	64.63	137,409	77.21	
Diabetes: Yes	21,140	59.39	72,290	40.62	<.0001
Diabetes: No	14,455	40.61	105,685	59.38	
Chronic lower respiratory diseases: Yes	28,392	79.76	114,759	64.48	<.0001
Chronic lower respiratory diseases: No	7,203	20.24	63,216	35.52	
Liver diseases: Yes	26,918	75.62	98,374	55.27	<.0001
Liver diseases: No	8,677	24.38	79,601	44.73	
Hypertensive diseases: Yes	23,823	66.93	89,480	50.28	<.0001

Hypertensive diseases: No	11,772	33.07	88,495	49.72	
**Psychiatric disorders (N(%))**					
Schizophrenic spectrum disorders: Yes	3,823	10.74	3,047	1.71	<.0001
Schizophrenic spectrum disorders: No	31,772	89.26	174,928	98.29	
Major depressive disorders: Yes	22,388	62.9	35,512	19.95	<.0001
Major depressive disorders: No	13,207	37.1	142,463	80.05	
Bipolar disorders: Yes	3,917	11	2,416	1.36	<.0001
Bipolar disorders: No	31,678	89	175,559	98.64	
Anxiety disorders: Yes	28,020	78.72	76,167	42.8	<.0001
Anxiety disorders: No	7,575	21.28	101,808	57.2	
Substance use disorders: Yes	2,994	8.41	2,790	1.57	<.0001
Substance use disorders: No	32,601	91.59	175,185	98.43	
Insomnia disorder: Yes	34,626	97.28	45,584	25.61	<.0001
Insomnia disorder: No	969	2.72	132,391	74.39	
Other mental disorders: Yes	13,978	39.27	26,654	14.98	<.0001
Other mental disorders: No	21,617	60.73	151,321	85.02	

The ZEG was defined as having a cumulative number of days of prescribed zolpidem exposure of 28 days or more within one year from the initial administration of zolpidem within the 12 years for the cohort. The ZNG who was not exposed to zolpidem during the study period. The control ZNG group was selected to match the ZEG by gender and age in a 1:5 ratio.
